# Ulcerative supraglottitis – a unique presentation of COVID-19 infection

**DOI:** 10.1007/s00405-025-09458-x

**Published:** 2025-05-17

**Authors:** Shmuel Wechsler, Jacob Pitaro, Nathan Shlamkovich, Limor Muallem Kalmovich, Haim Gavriel

**Affiliations:** 1 Department of Otolaryngology, Head and Neck Surgery, Shamir (Assaf Haroffeh) Medical Center, Tzriffin, Israel; 2 Innovation Center, Shamir (Assaf Harofeh) Medical Center, Zerifin, Israel; 3 Faculty of Medicine, Tel-Aviv University, Tel-Aviv, Israel

**Keywords:** Supraglottitis, Epiglottitis, COVID-19, Dysphagia

## Abstract

**Objective:**

COVID-19 has been previously considered a lower respiratory tract disease. However, as the disease has evolved, studies have described its upper respiratory involvement. This study's objective was to present, for the first time, a series of patients with supraglottitis with unique characteristics of coronavirus disease.

**Methods:**

This retrospective single-center case study included patients who were referred to the Emergency Department (ED) between June 1, 2022, and September 1, 2023, with clinical and endoscopic findings consisting of supraglottitis and positive PCR results for SARS-CoV-2.

**Results:**

Ten patients met the inclusion criteria. The mean age was 61. Six (60%) patients had comorbidities. Two (20%) patients were not vaccinated, whereas the others were vaccinated between one and five times. All patients presented with odynophagia, and most had fever and hoarseness. Laryngeal findings included epiglottic ulcers in the inner portion of the epiglottis in all patients, with varying degrees of severity. Presentation with a choking sensation, hoarseness, and elevated CRP and WBC levels may indicate a more severe disease course.

**Conclusion:**

The latest COVID-19 variants can manifest with clinical and endoscopic findings of acute supraglottitis, with specific endoscopic characteristics of the larynx. Physicians should be aware of this clinical entity, refer suspected patients to the ED, and perform urgent laryngoscopy, while the otolaryngologist should be aware of the specific endoscopic presentation and manage the patient accordingly.

**Level of evidence: 4:**

This study was approved by the institutional review board. Approval number- 0212-23ASF- 22/09/2023.

## Introduction

Coronavirus disease 2019 (COVID-19) is a highly contagious viral illness caused by severe acute respiratory syndrome coronavirus 2 (SARS-CoV-2). COVID-19 has had a catastrophic effect worldwide, resulting in more than seven million deaths [1]. The World Health Organization (WHO) declared it a global pandemic on March 11, 2020 [2]. Five SARS-COV-2 variants have been designated as variants of concern by the WHO and other regional agencies [3]: Alpha (B.1.1.7), Beta (B.1.351), Gamma (P.1), Delta (B.1.617.2), and the Omicron variant (B.1.1.529), first to be reported in South Africa in November 2021 [4, 5].

Previous studies have compared variations in transmissibility, clinical characteristics, severity, and outcomes between patients infected with early and late variants. In particular, studies have shown that late variants are characterized by higher transmissibility [6]. The severity of symptoms and clinical outcomes among variants has also changed slightly; the delta variant is associated with more severe symptoms, whereas the omicron variant appears to create less severe disease [7, 8].

COVID-19 was considered to be presented mainly as lower respiratory tract-related symptoms such as fever, cough, dyspnea, and chest tightness, which can progress rapidly to acute respiratory distress syndrome (ARDS) [9]. However, several articles have reported since 2021 that COVID-19-induced acute laryngitis requires emergency treatment, as indicated by acute epiglottitis in adults [10–14].

Iwamoto Et al. published a review of six cases of acute epiglottis caused by COVID-19; four of them required tracheotomy, and one patient was intubated [15]. These descriptive laryngeal findings are similar to those observed in patients with common acute epiglottitis. Only one report presented an image of acute epiglottis with the larynx partially affected by erosive lesions (N501Y type of alpha variant) [15].

Piersiala et al. described a case series of twenty young patients during the omicron wave in Sweeden in the last week of 2021 who were referred to the Ears, Nose, and throat (ENT) emergency department (ED) with odynophagia and severe throat pain. None of the patients described in this study presented with swollen or edematous epiglottis, and none required airway management. Only one patient presented with swelling of the arytenoid region, and the laryngeal findings in most patients included only general redness in the hypopharynx and larynx [16].

Meng et al. recently published a systematic review on acute epiglottitis with COVID-19. The authors identified 11 cases of this disease in the literature. Nearly half of patients required airway management (intubation or tracheotomy). The authors did not describe the laryngeal findings in these patients [17]. 

In this study, we describe the first series of patients with COVID-19 hospitalized with ulcerative acute infective supraglottitis (AIS) as their main presentation.

## Methods

### Design

We conducted a retrospective single-center case study. The inclusion criteria were patients who were referred to the ED at a tertiary Medical Center due to acute odynophagia and severe throat pain between June 1, 2022, and October 1, 2023, with laryngeal findings of supraglottitis and a confirmed COVID-19 diagnosis. In our Medical Center, all patients admitted between July 2020 and February 2023 were routinely tested using PCR for COVID-19, while since, only patients with suspected clinical manifestations have been tested for the virus. This study was approved by the institutional review board.

### Ethical considerations

This study was conducted in accordance with the ethical standards of our institution and in alignment with the 1964 Helsinki declaration and its subsequent amendments. The research received approval from the local Institutional Review Board (IRB Approval number: 0212-23 ASF, dated 22/09/2023). As determined by the IRB, this study did not require consent from participants due to the absence of direct interaction and the rigorous maintenance of confidentiality, achieved by omitting any identifying information from the data used. The authors declare that there are no conflicts of interest.

### Data collection

Medical records were reviewed, and patient demographics and medical histories were recorded.

Data on the following epidemiological variables were collected: sex, age, comorbidities, smoking status, and vaccination status for COVID-19 before current hospitalization.

The summary letter of the ED visit was reviewed for the onset of symptoms before the visit, including fever, cough, dyspnea, rhinorrhea, odynophagia, sore throat, hoarseness of voice, foreign body sensation, and choking sensation.

Medical records were collected during hospitalization. Laboratory findings, including leukocyte and neutrophil counts and C-reactive protein (CRP) levels, were obtained upon admission to the ENT department and during their stay if needed. Microbiological studies included blood cultures, throat swabs, and oropharynx PCR tests.

All patients had a laryngeal examination with flexible endoscopy recorded during their visit to the ED. Most patients had a follow-up laryngeal endoscopy, and the results were recorded.

The patients were divided into three subgroups according to disease severity and laryngeal findings.

Descriptive statistics were used to characterize the cohort.

## Results

The demographic and epidemiological characteristics of ten patients meeting the inclusion criteria were identified. The patients'ages ranged from 21 to 78 years, with a mean age of 61.20 (SD-17.83). There were five (50%) men. Six (60%) patients had comorbidities including diabetes and hypertension (Table 1). Three patients (30%) had either been active smokers or had smoked within the last five years. Eight (80%) patients were vaccinated with at least two doses of the Pfizer vaccine, and two patients were not vaccinated. The demographic data are summarized in Table 1.

**Table 1:** Epidemiological, clinical features, and vaccination status of supraglottitis with COVID-19 patients

Characteristics	Patients (*N* = 10)
Age- mean (SD)	61 (± 17)
Gender (f/m)	5 (50%)/5 (50%)
Comorbidities	7 (70%)
HTN	3 (30%)
DM	2 (20%)
IHD	1 (10%)
s/p RT	1 (10%)
S/p CVA	1 (10%)
Vocal cord polyp	1 (10%)
AF	1(10%)
Smoker	3 (30%)
Healthy	4 (40%)
Vaccination	
None	2 (20%)
2 vaccines	3 (30%)
3 vaccines	3 (30%)
4 vaccines	1 (10%)
5 vaccines	1 (10%)

*F* female, *M* male, *SD* standard deviation, *DM* diabetes mellitus, *HTN* hypertension, *IHD* ischemic heart disease, *AF* atrial fibrillation, *S/P* status post-RT- radiation therapy, *CVA* cerebrovascular accident

Smoker-smoker within the last five years with a minimum of 10 pack-years

**Fig. 1:**
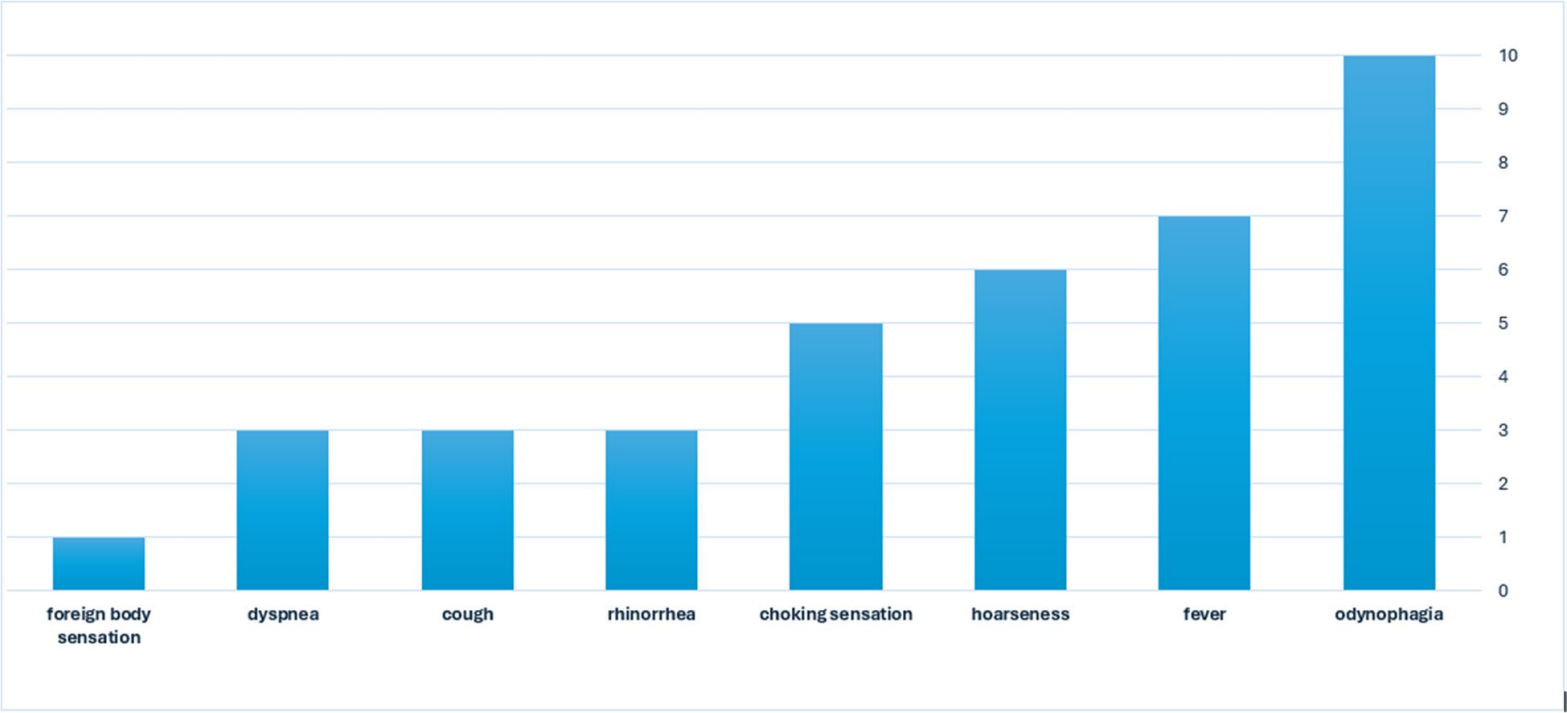
Patients' symptoms

All the patients had severe throat pain and odynophagia. Seven (70%) patients presented with fever, six (60%) with hoarseness, and five (50%) had a choking sensation (Fig. 1). The patients were referred to the ED with a mean time of 2.8 days (SD- 1.2) after the onset of symptoms. Laboratory tests revealed elevated CRP levels (mean, 80 mg/L, SD- 41), mildly elevated white blood cells (WBC; K/µL, mean, 10.69, SD- 3.9), and neutrophilia (K/micl mean, 8.29, SD- 8.29).

Regarding other coinfections, all patients had a positive PCR test for the COVID-19 virus and a negative blood culture test; nine (90%) patients had negative swab test results taken from their throat, and seven (70%) had negative PCR test results for other pathogens (Tables 2 and 3).

**Table 2:** Laboratory, laryngeal findings, treatment, and complications in patients with supraglottitis and covid 19

Age, sex	Vaccine status	Comorbidities or smoker	Days from Symptoms to ED	Signs & symptoms	Laboratory	Laryngocopic findings	Treatment	Days of hospitalization	Long term complication	Group
46, F	0	None	4	Hoarseness choking- sensation foreign body- sensation odynophagia cough fever	WBC-16.7 NEU-14.8 CRP-75 PCR-NEG SWAB-NEG BLOOD-NEG	Salivary accumulation epiglottic ulcer AEF ulcer Pus secretion Arytenoids edema	Amoxicillin\ sulbactam Steroids	5	None	3
78, M	4	Vocal cord. polyp, smoker	4	Hoarseness choking- sensation odynophagia fever	WBC-13.5 NEU-10.9 CRP-152 **PCR-HiB** SWAB- NEG BLOOD- NEG	Salivary accumulation epiglottic ulcer AEF ulcer Pus secretion	Amoxicillin\ sulbactam Steroids	5	None	3
76, F	3	HTN	2	Hoarseness odynophagia cough	WBC-7.8 NEU-5.5 CRP-39.3 PCR- NEG SWAB- NEG BLOOD- NEG	Salivary accumulation epiglottic ulcer AEF ulcer	Amoxicillin\ sulbactam Steroids	4	None	1
75, F	5	HTN, AF	1	Hoarseness choking- sensation odynophagia dyspnea rhinorrhea	WBC-15.7 NEU-11.9 CRP-150 PCR- NEG **SWAB-PA, CA** BLOOD- NEG	Salivary accumulation epiglottic ulcer AEF ulcer Pus secretion	Ceftriaxone Remdesivir Steroids	7	Laryngeal candidiasis	3
53, F	2	None	3	Odynophagia	WBC-5.9 NEU-4.9 CRP-17 **PCR- HiB** SWAB- NEG BLOOD- NEG	Salivary accumulation epiglottic ulcer AEF ulcer Pus secretion Arytenoids edema	Amoxicillin\ sulbactam Steroids	3	None	2
21, M	2	None, smoker	5	Odynophagia fever	WBC-4.9 NEU-3 CRP-45 PCR- NEG SWAB- NEG BLOOD- NEG	Salivary accumulation epiglottic ulcer AEF ulcer	Ceftriaxone Steroids	3	None	2
71, M	3	DM	2	Choking- sensation odynophagia dyspnea cough fever	WBC-12.1 NEU-9.7 CRP-77 **PCR-HSV** SWAB- NEG BLOOD- NEG	Salivary accumulation epiglottic ulcer AEF ulcer	Amoxicillin\ sulbactam Paxlovid Steroids	2	None	1
57, M	0	HTN, DM, s\p CVA, smoker	2	Hoarseness choking- sensation odynophagia dyspnea rhinorrhea fever	WBC-9.9 NEU-5.8 CRP-91.8 PCR- NEG SWAB- NEG BLOOD- NEG	Salivary accumulation epiglottic ulcer AEF ulcer Pus secretion Arytenoids edema	Amoxicillin\ sulbactam Steroids	3	None	2
62, F	3	S/P RT	2	Odynophagia fever	WBC-10.0 NEU-9.2 CRP-93 PCR- NEG SWAB- NEG BLOOD- NEG	Salivary accumulation epiglottic ulcer AEF ulcer Arytenoids edema	Amoxicillin\ sulbactam Steroids	6	None	1
73, M	2	IHD	3	Hoarseness odynophagia rhinorrhea fever	WBC-10.3 NEU-7.2 CRP-68.5 PCR- NEG SWAB- NEG BLOOD- NEG	Salivary accumulation epiglottic ulcer AEF ulcer Arytenoids edema	Amoxicillin\ sulbactam Steroids		None	1

The grouping of patients (groups 1,2, and 3) was based on the severity of their laryngeal findings

*F* female, *M* male, *SD* standard deviation, *HTN* hypertension, *IHD* ischemic heart disease, *DM* diabetes mellitus, *AF* atrial fibrillation, *S/P* status post, *RT* radiation therapy, *CVA* cerebrovascular accident, *NEG* negative, *AEF* aryepiglottic fold, *WBC* white blood count (K/ul), *CRP* C-reactive protein (mg/L), *NEU* neutrophils (abs; K/micl), *BLOOD* blood culture test, *SWAB* throat swab culture, *PCR* swab PCR test, *HiB* Hemophilus influenza type b, *HSV* Herpes simplex virus, *PA* Pseudomonas aeruginosa, *CA* Candida albicans

**Table 3:** Clinical data and treatment summary

		*N* (SD)	%
Days of hospitalization (mean)		3.40 days (± 1.65)	
Days from Symptoms to ED (mean)		2.80 days (± 1.20)	
Signs and symptoms	Odynophagia	10	100%
	Fever	7	70%
	Hoarseness	6	60%
	Choking sensation	5	50%
	Rhinorrhea	3	30%
	Cough	3	30%
	Dyspnea	3	30%
	Foreign body sensation	1	10%
Laboratory	CRP (mg/L; mean)	81.00 (± 43.98)	
	WBC (K/ul; mean)	10.68(± 3.90)	
	NEU (abs; K/micl; mean)	8.29(± 3.65)	
	PCR- NEG	7	70%
	PCR- HiB	2	20%
	PCR- HSV	1	10%
	Blood culture- NEG	10	100%
	Swab test- NEG	9	90%
	Swab test- PA + CA	1	10%
Laryngoscopic findings	Salivary accumulation	10	100%
	Epiglottic ulcer	10	100%
	Arytenoids edema	5	50%
	Pus secretions	5	50%
	AEF ulcer	4	40%
Treatment	Amoxicillin\sulbactam	8	80%
	Ceftriaxone	2	20%
	Single dose dexamethasone	9	90%
	Hydrocortisone (daily)	8	80%
	Remdesivir	1	10%
	Paxlovid	1	10%
Complications	None	9	90%
	Laryngeal candidiasis	1	10%

*SD* standard deviation, *NEG* negative, *AEF* aryepiglottic fold, *WBC* white blood count, *CRP* C-reactive protein, *NEU* neutrophiles, *PCR* polymerase chain reaction, *HiB* Hemophilus influenza type b, *HSV* herpes simplex virus, *PA* pseudomonas aeruginosa, *CA* candida albicans

The mean number of days of hospitalization was 3.4 days (SD- 1.6); all patients were treated with antibiotics and steroids, 8 (80%) with amoxicillin/sulbactam and two (20%) with ceftriaxone, nine (90%) with a single dose of 20 mg dexamethasone IV and eight (80%) with daily hydrocortisone. One patient (10%) was treated with remdesivir and one (10%) was treated with paxlovid.

No intensive care unit (ICU) admission or airway intervention was required. Vocal cord paresis was not observed during hospitalization. One patient experienced laryngeal complications during follow-up with long-term laryngeal candidiasis.

**Fig. 2:**
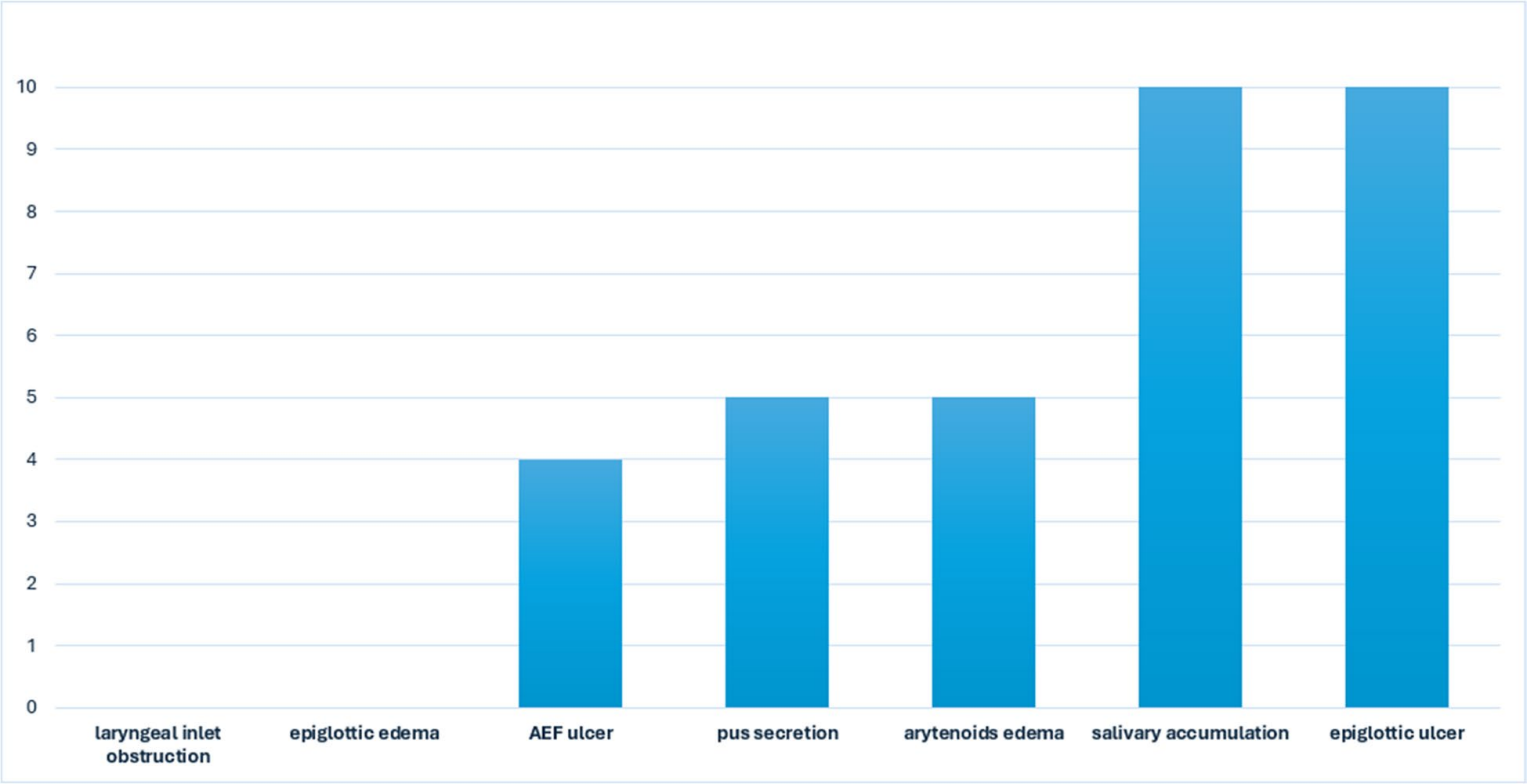
Laryngeal findings data. AEF- Aryepiglottic

The laryngeal findings included epiglottic ulcers in the inner portion of the epiglottis and salivary accumulation in all patients (100%); five (50%) patients presented with arytenoid edema, five with pus secretion in the larynx, and four (40%) had Aryepiglottic fold (AEF) ulcers. None of the patients had epiglottic edema or laryngeal inlet obstruction. (Fig. 2).

**Fig. 3:**
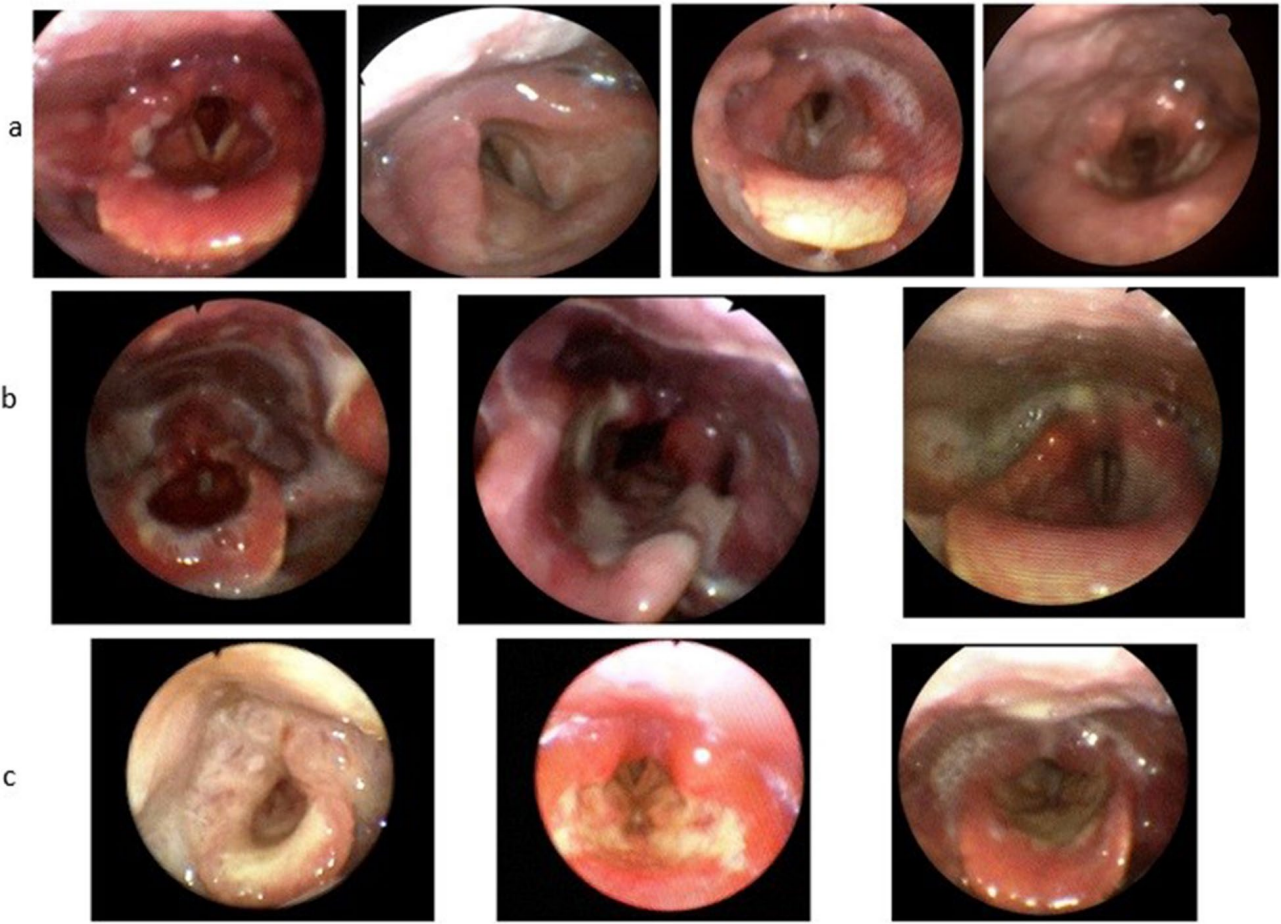
Laryngeal findings images. **a** Group 1: Local ulceration and mild edema. **b** Group 2: Widespread ulceration and mild edema. **c** Group 3: Ulceration and arytenoids edema

We divided the patients into three subgroups according to the severity of their laryngeal findings. Group 1 included patients with local ulcerations and mild edema (Fig. 3a). Group 2 included patients with widespread ulceration and mild edema (Fig. 3b). Group 3 included patients with ulcerations and more obvious edema, mainly in the arytenoid area (Fig. 3c).

We compared personal, clinical, and laboratory findings between the groups (Table 4). 1/3 of the patients in Groups 2 and 3 were smokers, whereas no smokers were included in Group 1. All patients in Group 1 had systemic comorbidities, whereas only 1/3 of the patients in groups 2 and 3 had systemic comorbidities. The mean vaccinations were 3.33 in Group 3, 1.33 in Group 2, and 2.75 in Group 1 (*p*-value: 0.379).

**Table 4:** Personal (a) clinical (b) and laboratory (c) differences in the laryngeal findings of the three Groups

**a-Personal**	**N**	**Gender (M:F)**	**Ages**	**Systemic comorbidities**	**Smoker**		**Number of Vaccines (mean)**	**Hospitalization Days (mean)**	
Group 1	4	2:2	62, 71, 73, 76	4/4	0		2.75	3.5	
Group 2	3	2:1	21, 53, 57	1/3	1/3		1.33	2	
Group 3	3	1:2	46, 78, 75	1/3	1/3		3.33	4.6	
**b-Clinical**	**N**	**Choking Sensation**	**Hoarseness**	**Fever**	**Foreign Body Sensation**		**Dyspnea**	**Cough**	**Rhinorrhea**
Group 1	4	1/4	2/4	3/4	0		1/4	2/4	1/4
Group 2	3	1/3	1/3	2/3	0		0	0	1/3
Group 3	3	3/3	3/3	2/3	1/3		1/3	1/3	1/3
**c-Laboratory**	**N**	**Other Pathogen**		**CRP**		**WBC**		**Neu**	
Group 1	4	1/4		69.4		10.05		7.9	
Group 2	3	1/3		51.2		6.9		4.5	
Group 3	3	2/3		125.6		15.3		12.5	

Personal (a), clinical (b), and laboratory (c) differences between the three subgroups revealed differences between Group 3 and other groups: clinically (choking sensation and hoarseness), laboratory results (other pathogens identified in throat, CRP, WBC, and Neu levels), and on hospitalization days

*WBC* white blood count (K/µL), *CRP* C-reactive protein (mg/L), *Neu* neutrophils (abs: K/micl)

A tendency towards a more severe disease course was observed in Group 3. All patients in Group 3 complained of choking sensation and hoarseness, in contrast to 1/3 in Group 2, and 1/4 and 2/4 in Group 1, respectively.

Co-infection in the swab test or PCR was found in 2/3 of the patients in Group 3 (1/4 and 1/3 in Groups 1 and 2, respectively). The mean CRP levels were higher in Group 3 (groups 3,2,1 CRP levels were 125, 51, and 69 (mg/L) respectively). WBC (K/ul) and Neutrophil counts (abs; K/micl) were 15.3 and 12.5 in Group 3, 6.9 and 4.5 in Group 2, and 10.05 and 7.9 in Group 1.

With regard to the length of hospitalization, Group 3 had the longest mean period of 4.6 days compared to 2 days in Group 2 and 3.5 days in Group 1.

## Discussion

### Covid-19

COVID-19 is currently a worldwide disease and probably will continue to be a seasonal disease similar to other viral illnesses [26]. As new variants of COVID-19 appear, symptoms may shift from the lower respiratory tract to the upper respiratory tract [16].

El-Anwar et al. evaluated the published literature on the ear, nose, and throat manifestations of early types of COVID-19 in 2020 and concluded that all manifestations were nonspecific and could be easily missed. No emergency ENT symptoms, including epistaxis or stridor, have been reported in patients with COVID-19. He concluded that ENT symptoms were uncommon compared with other upper respiratory viruses [27].

Later, in 2021, odynophagia was described during the omicron wave in Sweden, with only one patient described with AIS [16]. in this study, most patients with COVID-19 and odynophagia were young adults who were fully vaccinated.

To date, there have been only sporadic cases of supraglottitis caused by COVID-19 and the special characteristics of this manifestation of COVID-19 have not been defined [17]. In this study, we report for the first time a case series of COVID-19 positive patients with laryngeal findings of acute infectious supraglottitis (AIS) with special features, including epiglottic ulcers.

### Acute infectious supraglottitis (AIS)

AIS is a severe disease that can be life-threatening due to its risk of airway obstruction, with a reported rate of 8.8% of cases requiring airway intervention [18]. After the introduction of vaccination against *Haemophiles influenzae type B* (Hib), AIS is predominantly found in adults, and less acute course of supraglottitis is seen today [19]. The significant pathogens in acute epiglottitis (AE) post Hib-vaccine are very poorly defined, and main microbiologic finding in several studies is - S. pneumoniae, discovered only in approximately 10% of cases, leaving 90% with negative results [20].

Bacterial epiglottitis may cause airway obstruction, requiring airway intervention, such as intubation or tracheostomy, even on post-Hib vaccine era, but to a lesser extent [28].

The most consistent physical examination manifestation in all patients in our study was the presence of ulcers in the supraglottic area, mainly in the inner portion of the epiglottis. This laryngeal finding differs from the regular findings in AIS, which usually presents with widespread edema, and from AE, where a swollen red epiglottis is observed [28, 29].

In our study, none of the patients required airway management or ICU treatment, which may imply a milder disease course than that of bacterial AE. Likewise, the hospital stay was 3.4 days (SD 1.6), less than that described in the literature for bacterial etiologies of AE [29].

In our series, which took place during the omicron era, most patients were in their 70 s and were fully vaccinated. The rate of disease development in our cohort was similar to that of the classical AIS reported in the literature. In our study, the days from symptoms appearance to admission were 2.8 days (± 1.2 SD), similar to the duration of time described in other studies of AIS in the post-vaccination era of Hib [19].

### Treatment

Similar to other infectious etiologies of AE and AIS, all patients were treated with antibiotics and steroids, and no short-term complications were noted.

We decided to treat the patient with antibiotics, even though a viral pathogen such as COVID-19 was identified, to prevent and treat a possibility of bacterial co-infection. This decision was based on previous studies that stated that in post Hib vaccination-era most of AIS have an unidentified bacterial pathogen and probably viral etiology, yet recommending antibiotic treatment [19, 30, 31].

As PCR tests for viral pathogens are gaining popularity, the option of not treating patients with AIS and established viral pathogens should be considered in future studies. Not treating patients with viral AIS with antibiotics can save money in the future and prevent bacterial resistance. On the other hand, untreated patients with unrevealed bacterial causes can risk the airway of patients with infection in critical areas, such as the larynx.

### Subgrouping by laryngeal findings

In this study, we divided patients into groups according to the severity of their laryngeal disease on physical examination. Patients with a choking sensation, hoarseness, and higher CRP and WBC levels were more likely to have more severe disease and longer hospital stay. We found that the number of vaccines and the lack of systemic disease were not correlated with milder disease. A positive swab test PCR or other infectious etiologies led to a non-statistically significant severe course of the disease and were correlated with Group 3 (Tables 2 and 4).

This data should be interpreted with caution as the differences between the groups were not significant because of the small number of patients in each group.

### Supraglottic ulcers

Several systemic diseases can manifest as supraglottic ulcers. Autoimmune or inflammatory diseases, such as Crohn’s disease, Behçet's disease, granulomatosis with polyangiitis, pemphigus, and sarcoidosis, may present with laryngeal ulcers [21–23]. These etiologies are unlikely to be the etiology of laryngeal findings in our patients because the clinical manifestations were acute, with a single episode of ulcers in the epiglottis, similar to infectious non-recurrent disease. Furthermore, systemic inflammatory or autoimmune diseases usually present with systemic symptoms, whereas our patients were presented solely with upper respiratory tract symptoms that resolved later.

Other causes of adult supraglottitis that may appear with ulcers include the ingestion of corrosive substances or inhalation of hot vapors, smoke, or liquids [25]. These etiologies of supraglottitis were less likely to be the etiology of the ulcers in our study, as detailed anamnesis taken from the patients denied them.

Infectious diseases other than common bacteria may also cause laryngeal manifestations of supraglottic ulcers, such as tuberculosis, candidiasis, and HSV infection, with some cases requiring airway intervention [21, 24]. All patients in our study were tested for other infectious etiologies, and 60% of the patients had no microbiological evidence of pathogens other than COVID-19. In 40% of cases, we identified other pathogens using throat swab tests. However, their contribution to supraglottic diseases remains unclear.

There is a possibility that all patients in our study on patients with AIS presenting with epiglottic ulcers had another common etiology that we were unable to identify.

However, the similar appearance of the larynx in patients with COVID-19 likely implies a shared mechanism of COVID-19 in the larynx, which causes the same findings.

This study can be used by physicians to diagnose COVID-19 in patients with odynophagia by using this specific laryngeal manifestation.

## Conclusion

Supraglottitis is a novel manifestation of the latest COVID-19 variants. Its clinical course may share similar characteristics with those of other causes of supraglottitis; however, laryngeal presentation has unique characteristics. Physicians should be aware of the possibility of this clinical entity and consider referral to the ED for urgent laryngoscopy and treatment. The otolaryngologist should be aware of the specific endoscopic presentation and manage the patient accordingly. Further evaluation is required to identify the risk factors for more severe disease among patients.

## Abbreviations

COVID-19: Coronavirus disease 2019
ARDS: Acute respiratory distress syndrome
ED: Emergency department
ENT: Ears, Nose and throat
ICU: Intensive care unit
AIS: Acute infectious supraglottitis
AE: Acute epiglottitis
